# Herpes Zoster Induced Alveolar Bone Necrosis in Immunocompromised Patients; Two Case Reports

**Published:** 2016-09

**Authors:** Mahdi Gholami, Reza Shahakbari, Somayeh Abdolahpour, Masoud Hatami, Azam Roshanmir

**Affiliations:** 1 *Oral and Maxillofacial Disease Research Center, Dental Faculty, Mashhad University of Medical Science, Mashhad, Iran.*; 2 *Department of Oral and Maxillofacial Surgery, Dental Faculty, Mashhad University of Medical Science, Mashhad, Iran. *; 3 *Department of Oral and Maxillofacial Medicine, Dental Faculty, Mashhad University of Medical Science, Mashhad, Iran.*; 4 *Dentistry Research Center, Dental Faculty, Golestan University of Medical Science, Gorgan, Iran.*

**Keywords:** Herpes zoster infection, Osteonecrosis, Tooth exfoliation

## Abstract

**Introduction:**

Herpes zoster Infection (HZI) is a viral disease with painful skin rashes and blisters in a limited area on one side of the body, often in a strip. Osteonecrosis with spontaneous exfoliation of teeth in association with HZI of the mandibular nerve is a rare phenomenon. In this report, such an unusual complication of HZI is presented.

**Case Report:**

The clinical course of a 53-year-old woman and a 54-year-old man with HZI associated with alveolar bone necrosis and tooth exfoliation were reviewed in order to develop a patient profile for this rare combination of physical findings.

**Conclusion:**

In immunocompromised patients, the clinicians should consider HZI as a possible cause of tooth mobility, exfoliation, and alveolar osteonecrosis, which needs early intervention to prevent secondary complications.

## Introduction

Herpes zoster infection, commonly known as Shingles or Zona, is a viral disease characterized by unilateral painful skin rashes and blisters with linear pattern involvement. After the initial infection with Varicella zoster virus (VZV), which causes chickenpox in children and young people, the virus can become latent in the nerve cell bodies and less frequently in non-neuronal satellite cells of the dorsal root, cranial nerve or autonomic ganglion, without causing any symptoms (1,2). 

Years or decades after a chickenpox infection, the virus may break out of nerve cell bodies and travel down nerve axons to cause viral infection of the skin in the region of the nerve. The virus may spread from one or more ganglia along nerves of an affected segment and infect the corresponding dermatome causing a painful rash (3,4).

Although the rash usually heals within two to four weeks, some patients experience residual nerve pain for months or years, a condition called post herpetic neuralgia. Throughout the world the incidence rate of herpes zoster infection every year ranges from 1.2 to 3.4 cases per 1,000 healthy individuals, increasing to 3.9–11.8 per year per 1,000 individuals among those older than 65 years (5). Antiviral drug treatment can reduce the severity and duration of Herpes zoster infection if a seven- to ten-day course of these drugs is started within 72 hours of the appearance of the characteristic rash (5,6). The thoracolumbar trunk (especially T3 to L3) is most commonly affected (7). 

Herpes zoster infection (HZI) may affect cranial nerves and the trigeminal nerve is the most frequently affected (18.5%-22% of total cases). Oral manifestations appear when the second or third division is affected. Trigeminal nerve involvement is usually unilateral and limited to a single division, more often the ophthalmic (8). 

Less well recognized maxillofacial compli- cations include developmental anomalies such as irregular short roots and missing teeth, facial scarring, periodontitis, calcified and devitalized pulps, periapical lesions, and resorption of roots (9). Osteonecrosis with spontaneous exfoliation of teeth in association with HZI of the mandibular nerve is a rare phenomenon. In this report, such an unusual complication of HZI is presented. 

## Cases Report

**Fig 1:**
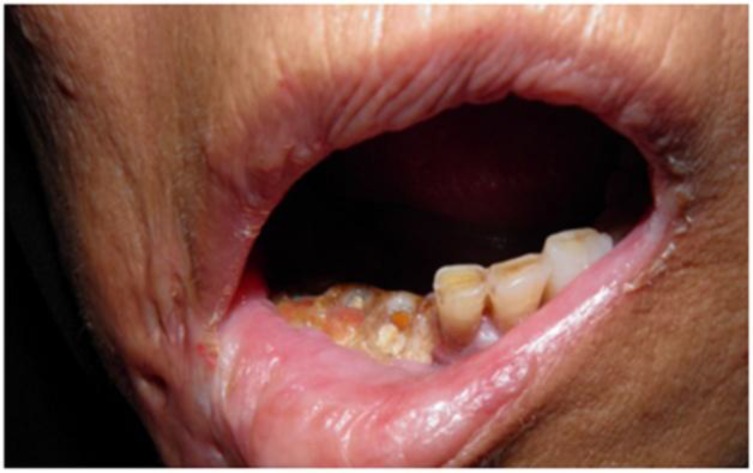
Osteonecrosis and spontaneous exfoliation of teeth involving right mandibular alveolar segment after herpes zoster infection

*Case1*: A 53-year-old woman, who had a kidney transplantation 9 months ago, was referred to the oral and maxillofacial surgery department, in the Shiraz Chamran hospital, complaining of a dull, throbbing pain in the right mandible and spontaneous tooth exfoliation (right lower incisor, lateral, and canine teeth).The patient’s medical history revealed that Herpes zoster infection had arisen 28 days after the transplantation, due to immunosuppressive therapy, in the third division of the trigeminal nerve. The patient had been treated with intravenous acyclovir and oral. Extraoral examination revealed facial and mucosal scar formation and hypopigmentation in the right side of the face restricted to the distribution of the mandibular division of the trigeminal nerve. Intraoral examination showed extensive necrosis of the buccal and lingual gingiva and exposure of the alveolar bone. The exposed bone was yellowish and the sockets of the exfoliated teeth were devoid of blood clots. The remaining teeth in the affected quadrant were mobile. (Fig.1) The panoramic radiograph showed a well demarcated sequestrum in the right mandibular alveolar segment. 

**Fig 2:**
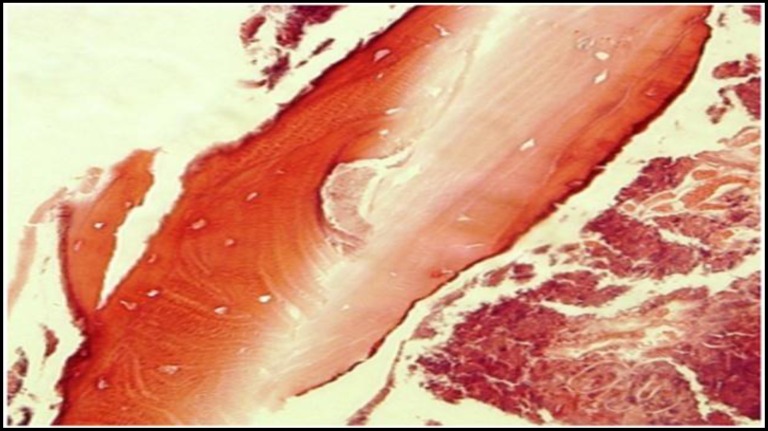
Necrotic bone trabeculae showing loss of osteocytes and surrounding bacterial colonization, 400X magnification

With the diagnosis of herpes zoster infection of trigeminal branch, under general anesthesia sequestrectomy of the necrotic bone was performed and the affected teeth were removed. As postoperative medication, ciprofloxacin 500 mg PO Q 12h and clindamicin 300 mg PO Q6h were prescribed for one week. Microscopic examination of the specimen showed eosinophilic, homogeneous non-vital bone tissue with peripheral resorption surrounded by reactive connective tissue. The osteocytic lacunae were empty. Intertrabecular spaces were filled by necrotic tissue and bacterial colonies (Fig.2). 

After sequestrectomy, healing of the surgical site was uneventful and the pain of the mandibular region was relived. At the regular evaluation of the patient, facial scar subsided and no complications were encountered.

*Case 2:*

**Fig 3:**
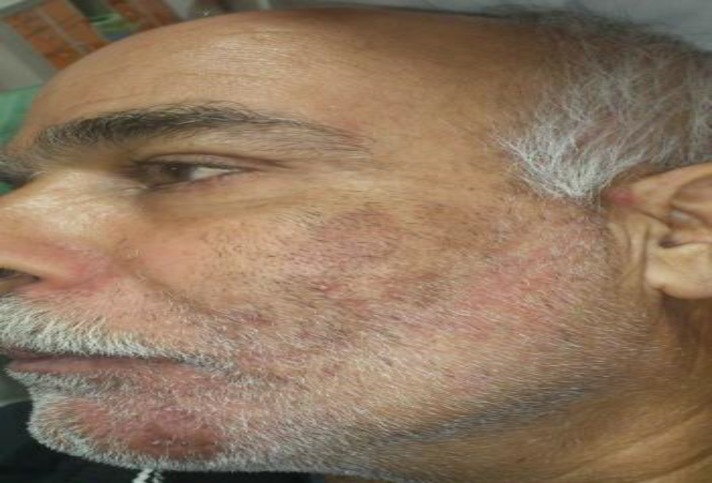
Patient extraoral view showing erythema and scar formation on the left side of the face

A 54-year-old man, who had a history of hypertension and uncontrolled diabetes for ten years, was referred to the oral and maxillofacial surgery department, in the Mashhad Dental School, for managing exposed necrotic bone around the remained roots of the left lower teeth. He declared that the left premolar teeth exfoliated spontaneously two weeks ago. The patient’s medical history revealed that herpes zoster infection had arisen a month ago, in the second and third division of the trigeminal nerve. The patient had been treated with intravenous acyclovir by an infectious specialist. Extra oral examination revealed erythema and scar formation in the left side of the face restricted to the distribution of V2 and V3 trigeminal nerve dermatomes (Fig.3).

**Fig 4:**
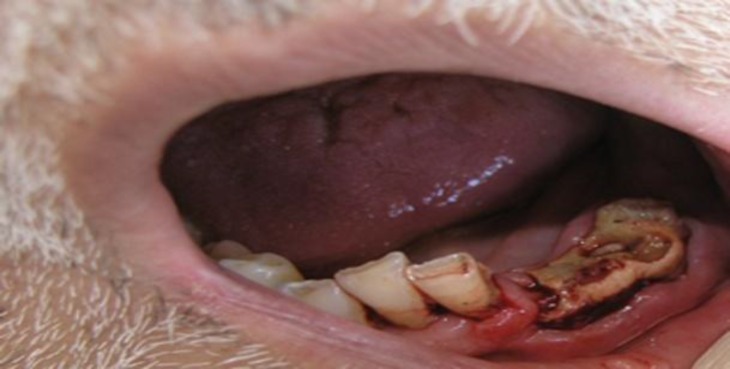
Intraoral examination, showing extensive necrosis of the buccal and lingual mucoperiosteum and exposure of the alveolar bone with remaining mobile roots in the affected quadrant

Intraoral examination showed exposed necrotic alveolar bone in the left side of the jaw. The remaining roots in the affected quadrant were mobile (Fig. 4).

**Fig 5:**
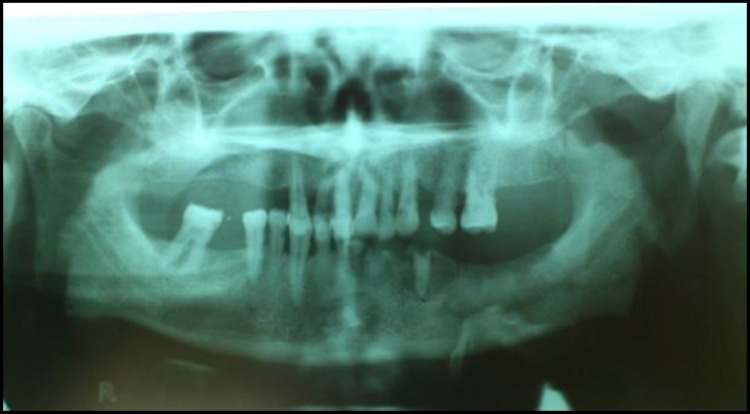
The panoramic radiograph demonstrates a well demarcated sequestrum and remaining mobile roots in the left mandibular alveolar segment

The panoramic radiograph showed a well demarcated sequestrum in the left mandibular alveolar segment (Fig.5).

**Fig 6:**
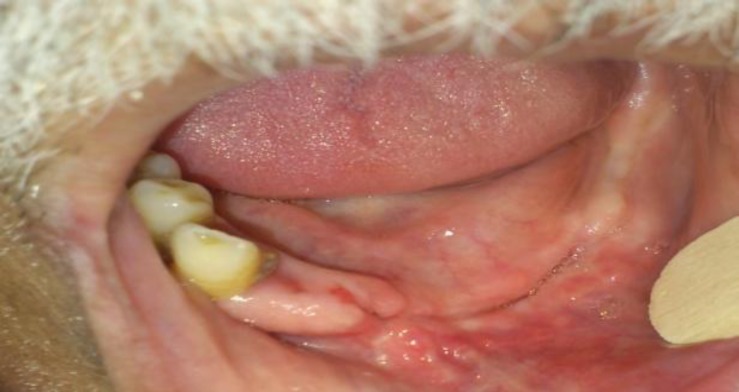
Postoperative intraoral view at one month follow up showed a healed surgical site. Right incisors were extracted because of periodontal involvement

With the diagnosis of Herpes zoster infection, under local anesthesia, sequestrectomy of the necrotic bone was performed and the affected roots were removed. As postoperative medication, clindamycin 300 mg PO Q6h was prescribed for one week. After sequestrectomy, healing of the surgical site was uneventful (Fig.6).

## Discussion

Maxillary and mandibular alveolar bone necrosis associated with Herpes zoster infection is rare. Up to 2009, postherpetic alveolar necrosis and spontaneous tooth exfoliation have been described in 41 patients (10). A review of the previously reported cases shows an age range between 6 and 85 years. Eight cases were under 40 years old, 10 were between 40 and 60 years, and 12 were above 60 years old with a mean age of 53.3 years (10). The increasing frequency of HZI with age has been suggested to be due to the disappearance of zoster-neutralizing antibodies which usually persist for 40 years after the original attack of chickenpox (7).

There is a slight predilection for post- HZI osteonecrosis is in the mandible (18 patients) compared to the maxilla (13 patients), and more lost teeth have been observed in the mandible compared to the maxilla (44 vs. 31). Of the lost teeth, 64 were anterior teeth and 61 were posterior teeth (1 study did not specify the location of the lost teeth) (11).

Fifteen patients had underlying disease including hematologic neoplasm, such as Hodgkin's disease, chronic hepatitis, Diabetes mellitus, Acquired Immunodeficiency Syndrome (AIDS), Tuberculosis, and *Immunosuppression* due to kidney transplantation. Herpes zoster was severe in all cases and the sites included the maxillary nerve in eleven patients, mandibular nerve in eighteen, ophthalmic and maxillary nerve in one, and maxillary and mandibular nerve in two. Maxillary and mandibular alveolar bone necrosis appeared 9–150 days after the onset of herpes zoster, with a mean of 30 days. Thirteen patients required the limited removal of teeth, while the others required widespread removal. The teeth in the affected segment exfoliated spontaneously in some cases (1-9). 

The pathogenesis of alveolar necrosis is still controversial. Two hypotheses are present. One possible explanation is ischemia. Vasculitis induced by the Varicella zoster virus may lead to necrosis of the periodontal tissue and alveolar bone (8,12). An alternative reason is that the alveolar artery may be compressed by the edema caused by the inflammation of the alveolar nerve in the narrow maxillary or mandibular canal. This process may result in ischemia and subsequent necrosis of the periodontal tissue and alveolar bone (3,7). In addition, pre-existing pulpal and periodontal infection may contribute to the mechanism (12).

Another explanation is bacterial invasion through blistering and acantholysis of the mucosa in the affected region caused by VZV. In an immunocompromised patient a durable mucosal ulcer provides perfect gate for actinomycete and staph variants to invade, reside, and form osteomyelitis. 

Jaw osteonecrosis clinically appears as denudation of bone with exposure of teeth sockets. Panoramic radiographs may show empty teeth sockets and sequestrations of necrotizing bone and rarely a “moth-eaten” appearance of the underlying bone (13). Recently, numerous laboratory diagnostic methods have been developed for the diagnosis of herpes zoster infection. These include dot-blot hybridization, polymerase chain reaction, and direct staining of cytologic smears with fluorescent monoclonal antibodies for VZV. Along with these, histopathological examination of the necrotizing alveolar bone has been proposed (13).

## Conclusion

In conclusion, herpes zoster infection is a rather common viral infection with a wide range of manifestations especially in the oral cavity. In immunocompromised patients the clinicians should consider it as a possible cause of tooth mobility, exfoliation, and alveolar osteonecrosis, which needs early intervention to prevent secondary complications.
